# The effect of luteal phase gonadotropin-releasing hormone antagonist administration on IVF outcomes in women at risk of OHSS

**Published:** 2016-08

**Authors:** Maryam Eftekhar, Sepideh Miraj, Zahrasadat Mortazavifar

**Affiliations:** 1 *Research and Clinical Center for Infertility, School of Medicine, Shahid Sadoughi University of Medical Sciences, Yazd, Iran.*; 2 *Department of Obstetrics and Gynecology, School of Medicine, Shahrekord University of Medical Sciences, Shahrekord, Iran.*

**Keywords:** *Gonadotropin-releasing hormone antagonist*, *Pregnancy outcome*, *In vitro fertilization*

## Abstract

**Background:**

Gonadotropin-releasing hormone (GnRH) plays essential roles in embryo implantation, invasion of trophoblastic tissue, and steroid synthesis in the placenta.

**Objective:**

The aim of this study was to evaluate the effect of GnRH antagonist administration on pregnancy outcomes in early implantation period.

**Materials and Methods:**

In this retrospective study, 94 infertile women undergoing GnRH antagonist protocol who were at risk of ovarian hyperstimulation syndrome (OHSS) were included. Sixty-seven patients (group I) received Cetrorelix 0.25 mg/daily in the luteal phase for 3 days while in 27 participants (group II), it was not administered. Pregnancy outcomes were assessed based on chemical and clinical pregnancy rates.

**Results:**

The pregnancy outcomes were not significantly different between two groups (p=0.224).

**Conclusion:**

The present study proposed that luteal phase GnRH antagonist administration does not influence the chance of successful pregnancy outcomes.

## Introduction

Female genital tissues like ovary, endometrium, and placenta are extra pituitary tissues which express Gonadotropin-releasing hormone (GnRH) receptors (1). GnRH plays essential roles in embryo implantation, invasion of trophoblastic tissue, and steroid synthesis in the placenta (2). There are limited evidences of GnRH antagonists’ effects on pregnancy outcomes. Several protocols such as GnRH antagonist protocol were used for in vitro fertilization (IVF) (3, 4). Previous studies evaluated the role of GnRH antagonist in women with poor response to ovulation stimulation. Some studies were demonstrated GnRH antagonist is comparable with GnRH agonist (5, 6). The most important GnRH antagonist benefits are including: decrease the need of exogenous gonadotropin, shorter time for stimulation, and a cost effective protocol (3, 4, 7-9). 

Also GnRH antagonist causes the regression of established severe ovarian hyper stimulation syndrome (OHSS) by luteolysis as a key mechanism in prevention of OHSS (2). After introduction of GnRH antagonist into clinical practice, it reduced OHSS rate in IVF/ICSI cycles (9). GnRH antagonist can improve the poor response to ovulation stimulation (6, 10, 11). Although many studies showed the benefits of GnRH antagonist on IVF/ICSI cycle outcomes but its effect is controversial. The use of GnRH antagonists is generally limited to the last few days of ovulation in IVF/ICSI cycles. 

The aim of this study was to evaluate the GnRH antagonist effects at pharmacological doses given in early implantation period on pregnancy outcomes.

## Materials and methods

In this retrospective study, medical records of 94 women that were at risk of OHSS in IVF/ICSI cycle and has been reffered to the Research and Clinical Center for Infertility, Yazd, Iran between October 2014 and February 2015 were reviewed. The study protocol was approved by the ethics committee of the Research and Clinical Center for Infertility, Yazd, Iran and oral consent was obtained from all participants. 

Inclusion criteria were women under 40 years old, having more than 20 follicles (>14 mm) at triggering time, and on risk of OHSS during ICSI-IVF cycle with embryo transfer. Women with history of endometriosis, history of more than 2 implantation failure, and severe male factor were excluded. 

Totally, 94 eligible women were studied in two groups. All participants were treated with GnRH antagonist protocol. Patients received recombinant human follicle stimulating hormone (Gonal-F) (150 IU, subcutaneously) for 5 days. Serial trans-vaginal sonography was performed. When the mature follicle (≥14 mm) was detected, GnRH antagonist (Cetrotide) (0.25 mg/daily, subcutaneously) was injected. Triggering was started with 1500 IU hCG (Pregnyl, Organon, Netherland) and 0.2 mg GnRH-a (Decapeptyl^®^, 0.1 mg) (subcutaneously) injection when at least two follicles with a mean diameter of 17 mm was observed. 

Trans-vaginal egg retrieval was done under sedation after 36 hrs. 67 women received 25 mg Cetrotide subcutaneously for 3 days from day of oocyte retrieval (case group) and 27 participants did not receive Cetrotide in luteal phase (control group). 2 embryos were transferred 48 hr after oocyte retrieval using an embryo transfer Labotect catheter (Labotect Gmbh. Lbabor-Technik-Göttingen GmbH, Gottingen, Germany) In all patients. All transferred embryos were in grade A and B. Progesterone suppositories (Cyclogest®) 400 mg twice in a day was used vaginally on the day of oocyte collection for luteal phase support, and it continued until the fetal heart activity was documented by ultrasonography. Serum beta-hCG (β-hCG) was assessed on day 14 after embryo transfer.

Positive pregnancy test was define as β-hCG >50 IU/L. Pregnancy outcomes were assessed based on clinical pregnancy (observation of fetal heart activity by transvaginal ultrasonography 2-3 wks after positive β-hCG). Implantation rate was defined as the ratio of gestational sacs to the number of embryos transferred.

**Statistical analysis**

All of statistical analysis was done by SPSS 20 (SPSS, Chicago, IL). The normal distribution of data was checked. Mean±SD were calculated for descriptive analysis. Independent t-test and ^2^ were used. The statistical significances considered as 0.05. According with power analysis the power of study was 0.8 and α was 0.05.

## Results

The mean age of participants was 28.56±4.03 yrs in case and 28.03±4.8 yrs in control group. Basic characteristics of participants in groups are shown in table I. There were not statistically differences in age, duration of infertility, basal FSH serum, progesterone and estradiol level in the day of HCG triggering between groups. While the mean number of embryo was different (Table I). The pregnancy outcome was not significantly different between case and control group (p=0.224). The implantation rate was 14.39% in case group and 9.25% in controls (p=0.089) (Table II).

**Table I:** The basic characteristics of patients in two groups

**Variables**	**Case group (n=67)**	**Control group (n=27)**	**p-value** *
Age (year)	28.56 ± 4.03	28.03 ± 4.8	0.593
3^rd^ day FSH level	5.76 ± 2.73	5.71 ± 3.42	0.942
Infertility duration (year)	6.73 ± 3.85	6.66 ± 4.49	0.942
Serum progesterone** (ng/ml)	1.09 ± 0.55	1.11 ± 0.55	0.924
Serum estradiol** (pg/mL)	3337.07 ± 514.44	3301.63 ± 459.04	0.756

Data are presented as mean±SD.

* Independent Students’ *t*-Test

** in the day of HCG triggering

**Table II:** ART outcomes in two studied groups

	**Case group (n=67)**	**Control group (n=27)**	**p-value** *
Number of oocyte a	19.86 ± 4.94	18.51 ± 2.84	0.023
Number of embryo a	6.31 ± 5.21	5.22 ± 4.06	0.332
Clinical pregnancy rate b	19 (28.78%)	5 (18.51%)	0.224
Implantation rate	14.39%	9.25%	0.089

a: Data are presented as mean±SD.

b : Data are presented as n(%).

* ^2^ test

## Discussion

Our results showed that luteal phase GnRH antagonist administration did not influence the chance of pregnancy. The clinical pregnancy rate in studied groups was not significantly different. Triggering of final oocyte maturation by hCG induces massive luteinization, increase angiogenic factors secretion (such as angiotensin II, interleukins, vascular endothelial growth factor, histamine, prolactin, prostaglandins, endothelin-1, and selectins) from corpus luteums of hyperstimulated ovaries. It leads to development of OHSS by increase in vascular permeability, and finally fluid shift to the third space (12-15).

Previous studies reported that GnRH antagonist administration in the luteal phase improved severe OHSS in two days after injection of GnRH antagonist by decreasing the ovarian volume, hematocrit, ascites, and oestradiol and progesterone concentrations (3, 4, 8). It suggests a luteolytic effect of the GnRH antagonist that lead to a decrease of ovarian activity and angiogenic factors secretion, resulting in regression of severe OHSS (4, 8). “GnRH antagonist inhibits Matrix metalloproteinase (MMP) and therefore can disrupt the implantation. GnRH antagonist effect on the expression of *HOXA10* genes in endometrium which is an important regulator of endometrial receptivity” (16).

GnRH antagonist administration during peri-implantation period may cause some concern about the potential adverse effects of GnRH antagonist on embryo implantation, pregnancy and neonatal outcomes (11). Our findings showed that pregnancy rate is similar between two studied groups. There are few studies about the effect of luteal phase GnRH antagonist administration on pregnancy outcomes (8, 11, 12). Lainas *et al* in a prospective cohort study on 192 IVF patients who were at risk of OHSS showed that pregnancy and neonatal outcomes did not decrease after luteal GnRH antagonist administration (1). Some recent studies documented that GnRh antagonist administration is not associated with pregnancy or congenital adverse effects (1-4, 8-11, 17-20). 

As our study limitations, OHSS evolution, neonate and children outcomes were not studied. Also the follow up period was very short. 

## Conclusion

In conclusion, luteal phase GnRH antagonist administration does not influence the chance of pregnancy after ART. The incidence of chemical and clinical pregnancy in groups was not significantly different.
